# Children’s Imaginaries of Human-Robot Interaction in Healthcare

**DOI:** 10.3390/ijerph15050970

**Published:** 2018-05-12

**Authors:** Núria Vallès-Peris, Cecilio Angulo, Miquel Domènech

**Affiliations:** 1 Department of Social Psychology, Universitat Autònoma de Barcelona, 08193 Barcelona, Spain; nuria.valles@uab.cat; 2 Automatic Control Department, Universitat Politècnica de Catalunya, UPC BarcelonaTech, 08028 Barcelona, Spain; cecilio.angulo@upc.edu

**Keywords:** imaginaries, social robots, children’s hospital, participatory process, ethics of care

## Abstract

This paper analyzes children’s imaginaries of Human-Robots Interaction (HRI) in the context of social robots in healthcare, and it explores ethical and social issues when designing a social robot for a children’s hospital. Based on approaches that emphasize the reciprocal relationship between society and technology, the analytical force of imaginaries lies in their capacity to be embedded in practices and interactions as well as to affect the construction and applications of surrounding technologies. The study is based on a participatory process carried out with six-year-old children for the design of a robot. Imaginaries of HRI are analyzed from a care-centered approach focusing on children’s values and practices as related to their representation of care. The conceptualization of HRI as an assemblage of interactions, the prospective bidirectional care relationships with robots, and the engagement with the robot as an entity of multiple potential robots are the major findings of this study. The study shows the potential of studying imaginaries of HRI, and it concludes that their integration in the final design of robots is a way of including ethical values in it.

## 1. Introduction

In recent decades, robots have been gradually incorporated into the social environments of our daily lives to perform duties traditionally assigned to humans, in what was an unexpected scenario until recently. Designed to interact with people in a manner consistent with human psychology and following the guidelines and rules of social interaction [1], social robots have been developed for several applications, among them pediatric hospitals. Interactions with social robots have been designed to reduce pain and anxiety when children with chronic illnesses are subjected to several kinds of specific interventions or regular treatments, and to help make their hospital stay more pleasant [2]. The rapid development of social robots has also kindled the emergence of an important topic of research that of what constitutes successful human-robot interaction. Research on Human-Robot Interaction (HRI) aims to understand the interactions between humans and robots and build systems to support such interactions [3]. Within the study of HRI, interaction between children and robots has a special place, as children have different behavioral patterns from adults. Some scholars have referred to this field of study as Children-Robot Interactions (cHRI) [4]. Research on cHRI demonstrates that the interaction between robots and children can prove highly effective in healthcare, especially in robot-assisted therapies [4,5], and in the use of robots in treatment of those with autistic spectrum disorders [6]. Although assessing cHRI is especially difficult if compared with adult robot interactions, it has been demonstrated that children, particularly young children, have the ability to engage with robots and treat them as lifelike agents, because children anthropomorphize them in their pretend play [4]. However, beyond the technological developments involved in the alleged progressive incorporation of robots in healthcare and in increasingly complex HRI systems, new ethical, political, and social challenges arise [7] when we seek to address the issue of how social robots and HRI improve—or reduce—children’s well-being [8].

Artifacts and technologies have been used for some time in hospitals and healthcare environments. However, the use of social robots has opened up new controversies (or has revived old ones, though in new forms). The evocative potential of robots, which make us reflect about our own humanity [9], has led to the emergence of new social and ethical debates concerning their adequacy or their desirability, especially when they are used with children and other vulnerable collectives. Some voices warn us about the reduction of human contact [10] and the negative impact that spending too much time in the company of a robot could have for children, as it could interfere with the infant’s learning about the give-and-take of human care relationships as well as with their cognitive and linguistic development [11]. Others advocate for the design of moral machines capable of making ethical decisions, giving robots ethical principles to guide their behavior and select the best action at each moment [12]. Beyond both hopeless and hopeful scenarios regarding the use of robotics in our daily lives, this paper proposes the analysis of children’s imaginaries of HRI as a way to approach possible social and ethical controversies about the use of social-care robots in particular contexts and, more specifically, in the context of children’s hospitals.

In 2016, a participatory process with six-year-old children—together with an interdisciplinary research team of roboticists, medical staff and social scientists—was carried out in a school in order to facilitate the design of robots that might be appropriate for hospital settings. For the analysis of this experience of “design in the wild” [13], we took an approach complementary to contemporary research in social robotics, which is mainly focused on technical aspects and “erases the social” [14]. It was with this intention that we chose to analyze the children’s imaginaries of Human-Robots Interactions (HRI) emerging in the context of the design process. The use of imaginaries as analytical objects was based on two assumptions: first, that the role of imaginaries and expectations was embedded in the practices and interactions of the children involved in the design process; and second, that the imaginaries surrounding robots affect their construction and application.

The data obtained during the process were analyzed from a care-centered approach, using perspectives and debates that made it possible to focus on children’s values, norms and practices with relation to care. Thus, the focus of our analysis was on HRI, on the interactions between humans and robots, which contain prospective imaginaries of relationships, planned and enacted in practices and particular contexts.

### 1.1. Imaginaries and Participation

Following the conceptualization of the significations of the imaginary developed by Castoriadis [15], many disciplines have taken the “imaginary” as an analytical object. “Social imaginaries” can be understood as frameworks that individuals create in order to constitute their practices and social surroundings and that account for the existence of a society’s common practices or conditions of politics, economics and so on [16,17]. In the use of imaginary significations in technology, the underlying assumption is that the imagination is a social practice deployed in the production of science and technology, being the creation of future imaginaries a major part of techno-scientific work [17]. Imaginary signification is not inherent to technological artifacts, but it is related to their context and intertwined in complex processes of negotiation of a social order [18]. In the field of science and technology studies (STS), the imaginary has been used for evoking visions, scenarios and expectations about the future as well as about the rise and/or fall of various scientific and technological fields [19]. STS scholars have demonstrated that these imaginaries are embedded in the practices and the organization of technoscience [17,20,21] and, at the same time, they inform and shape the trajectories of research and innovation [22]. Imaginaries are not only linked to particular research or innovation projects, they are almost always imbued with an implicit understanding of the social world—for instance, of how technology can serve public needs and what could be good or bad for society [23,24]. Thus, technological development cannot be understood as something pre-determined in a social or technological context [19], but it is something performed through a process of negotiation of imaginary significations and influenced by socio-political, economical and technoscientific conditions.

As Fujimura [17] has shown, creating future imaginaries is a major part of the work carried out by those involved in robotics research and development. However, in the daily dynamics of production and innovation, social robots are ideated and developed far from the spaces where they are to be applied. In the same manner, the process of creating social robots is in the hands of a very small group of people (university, corporate, governmental researchers, and funding agencies), who decide what constitutes a valuable research project and what the needs of society are [25]. These groups of actors work with the imaginaries present in future-oriented discussions, where the expectation is that robots will become part of our everyday life, working alongside humans as assistants, caregivers or therapeutic supporters. People that develop social robots represent them as technological solutions for social problems that are not exclusively technical [26]. In the case of social robots developed for children’s hospitals, telepresence robots provide social interaction for hospitalized children [27]; socially assistive robots help children to follow their dietary of therapeutic regimes [28], or reduce pain and anxiety when children are subjected to any kind of intervention or treatment [29,30]. But what are the imaginaries of the final users of robots for children’s hospitals? How do children themselves represent their interaction with robots?

Instead of a technocentric approach presuming that the public has to inevitably adapt to the integration of social robots [25] instead of shaping them [31], some authors [14,17] propose a different conceptualization of the relations between humans and machines. These approaches analyze “how social and cultural factors influence the way technologies are designed, used and evaluated, as well as how technologies affect our construction of social values and meanings” [25] (p. 1). On the basis of this reciprocal and dynamic relationship between technologies and society from the initial stages of design [32,33,34,35] it is relevant to inquire about children’s imaginaries about HRI with robots designed for taking care of them. To do this, we collected the proposal of some scholars [25,35] for using a range of methodologies of participatory design to support the participation of multiple stakeholders and disciplines in the development of social robots.

Parallel to a debate that demands greater public involvement in assessing the risks and uncertainties of new technologies [36,37,38,39], in the area of computer sciences and engineering the logics of participation [40,41] and user involvement [42] has also gained prominence. Although participatory episodes with children are not very common [37], in the field of Human-Computer Interaction, particularly in the area of Interaction Design and Children in computer science, there has been extensive development of methods and techniques for involving children in the design of new technology [42]. This kind of approaches understands children as an important group of technology users and consumers, which implies the need to enquire about ways to develop new technologies that respect them and respond to their needs [43]. They depart from the general philosophy of user-centered design, which has as its principal component that users, or people who will eventually use the technology, must be involved at some point in the design process [44]. In this sense Druin [43] has developed a popular model in order to understand the several roles—as users, testers, informants or design partners—that children can adopt in a participatory design process and the ways these roles can affect the produced technologies. Once we regard children as users or consumers, the more they participate in the early design stages, the more the development of innovations and improvements to the final design of technology is facilitated by them [44,45,46]. Also in healthcare, there are interesting experiences which take up this tradition and use participatory methodologies for the design of prototypes in order to ensure the inclusion of children’s own views and experiences, for example, in improving the design of children’s prostheses [47].

### 1.2. Care in HRI with Children

Fisher and Tronto [48] define care as “everything we do to maintain, continue and repair our world so that we can live in it as well as possible. This world includes our bodies, ourselves and our environment, all that we seek to interweave in a complex web of sustaining life” [48] (p. 30). The ethics of care contains two basic dimensions [49,50]: A first dimension refers to care as a series of concrete activities, i.e., care in its most everyday sense of “caring for” and “taking care of“. The second one refers to the set of values that guide action in various social spheres.

In our research, the first dimension is clearly materialized in the artifact to be designed by children, a social robot for taking care of children in a hospital. A robot that is used in the care of people in general is called a care robot [35]. Care robots are designed for use at home, in hospitals, or in other settings in order to assist in, support, or provide care for the sick, disabled, children, elderly or otherwise vulnerable persons [51]. The tasks to be performed by care robots can be classified into: providing assistance in care-giving tasks (feeding, bathing); monitoring a patient’s health status; and/or providing social care or companionship [11]. In our experiment, the social robots to be designed by children could be included in the last category, because these robots are specially characterized by their flexible definition according to context, users and task to be performed.

As for the second dimension, we have incorporated the debates around an ethics of care as a way of articulating the analysis of children’s imaginaries of HRI when designing a social robot for healthcare contexts. One of the underlying motivations of taking an ethics-of-care approach is to focus attention on a subject living in a network of relationships in which each individual has to reconcile different forms of caring responsibilities [52]. In healthcare, the ethics of care provided the ground for challenging both the ideal of an autonomous subject in the medical context and its principle-based medical ethics, which often is organized around four principles: autonomy, beneficence, non-maleficence and justice [53]. The ethics of care, with its focus on caring relations, challenges the idea of autonomy [53] and shows that good care does not have as much to do with the ideal of choice—dominant in healthcare—as with particular daily practices of care [54]. Following this care approach, the concept of autonomy is reformulated [55], questioning the idea of self-sufficiency and independence and proposing a relational autonomy which assumes that subjects are always in relation to others. Besides this, in the case of children, some critical perspectives in the field’s philosophical reflection highlight the pernicious consequences that a discourse of autonomy may have for children [56]. As Arneil [56] puts it, if we take seriously children’s need for care, then we move from a focus on the liberal right of autonomy to a re-conceptualized understanding of the need (and responsibility) for care.

We propose the opening up of new perspectives in the analysis of multiple caring interdependency relations as a radical political view on the analysis of imaginaries in robotics. In this paper, the analysis of children’s imaginaries of HRI in a healthcare context is primarily carried out from an ethics of care approach. HRI, more than the social robot itself, contains the ethics of care approach in a nutshell. Of course, children-robot interactions are linked to the robot’s materiality—its attributed features and functionalities—in such a way that all potential caring interactions are enacted in the robot’s materiality. As has been demonstrated by studies about user relations and biographies of technology [21], working on practical solutions and elaborating ideas, concepts and prototypes is crucial for the canalization of imagination. With this in mind, the main focus in the analysis of imaginaries is not on the designed robots, but on caring interactions between humans and robots, which contain a prospective network of relationships which are planned and enacted in particular practices and contexts.

## 2. Methods

The primary aim of our study was to produce a methodology in order to develop a participatory process to design a social robot for children’s healthcare. For the purpose of this paper, children’s imaginaries of HRI were analyzed in the process of study. Inspired by the method of “thick description”, we used participant observation throughout the experience to produce a narrative description of imaginaries of HRI. All sessions were video-recorded, and a field diary was kept on a regular basis. Thick description is a qualitative approach used in anthropology, sociology and psychology that goes beyond facts and appearances to include details, context, emotion and the webs of social relationships. Thick description inserts history into experience, establishing the significance of an experience or a sequence of events [57]. It emphasizes the interpretative aspects of description rather than detail per se, and this is what makes it a “thick” (as opposed to a “thin”) description [58]. What this paper presents is an in depth description of the process of designing of a social robot with children. The practices, actions and events that took place in the school during the participatory process are interpreted as assigning purpose and intentionality to them [58]. The explicative and interpretative tasks are organized around the children’s process of materially configuring a social robot. The process of drawing, building and playing with prototypes, as well as the drawings and the prototypes elaborated by children, are themselves the primary sources of the analysis which enables us to produce a narrative description of the children’s imaginaries of HRI.

### The Participatory Process

The process for designing a social robot with children in a school in order to facilitate designs for robots that might be appropriate for a hospital setting was planned together by an interdisciplinary team in the context of a wider project to develop an innovative health program for a children’s hospital. The project team included the innovation department at the hospital, a team of roboticists and a team of social scientists. Three work sessions were held with the whole project team in order to design the workshops that would be conducted at the school. According to the discussions held by the team, the design process was systematized into six phases; each phase was further defined in relation to an objective to be reached while working with children in a set of workshops (Table 1). Some objectives required only one work session, while others took more than one. After this initial planning of the project, all the workshop activities were specified in fuller detail together with the two teachers of robotics, in an attempt to use interesting dynamics and methodologies properly adapted to six-year-old children.

The project was completed in a school during a three-month period, and consisted of twelve weekly 50-min workshops. In most of the workshop sessions, instead of working with the whole group, subgroups of five to six pupils were organized to facilitate the participation of all children.

The experience took place at a school covering pre-school to Baccalaureate grade levels. The institution is a semi-private school of middle upper class pupils with two classes per grade level. The school is situated in Barcelona (Spain). It uses innovative pedagogies based on the theory of multiple intelligences and, as a distinctive relevant feature of its educational project, it includes the learning of robotics from the early stages of education. To make this possible, the school has two teachers of robotics for pupils six to twelve years old. The participatory process was carried out together with the teachers of robotics during their lessons’ timesheet. In the school, there is a specific room for the robotics class, where children up to seven years old have their lessons and where all the robotics materials are kept. However, the school organizes the first robotics lessons for younger children in their ordinary classroom, were teachers bring the necessary materials for each lesson. This is the reason why the participatory process with six-year-old children was held in their regular classroom, not in the robotics one.

One first-grade class with thirty 6-year-old pupils participated in the experience. A total of twelve workshops were carried out in the school. All the workshops were participated by the children, two teachers, two social scientists and two roboticists. All the parents of the children involved in the process gave informed consent for their son or daughter to participate in the study. They also authorized the researchers to record and analyze the experience, and to compile and analyze all the materials produced during the process. The following is the description of the activities carried out (Table 1):

- *Phase 1. Organizing an interdisciplinary team.* In one session, the project and the team were presented to the children. The goal was to let the children know about the activities and tasks of roboticists to help them assume the role they were asked to adopt. An activity was organized in which children defined what an engineer that makes robots is or does. Based on their definitions, a discussion followed where their teachers of robotics and the roboticists in the research team explained their work to the children. The presentation of a roboticist’ work was based on the specific example of designing a social robot for a children’s hospital. Children were told that the roboticists needed their help to include their perspectives as stakeholders.
- *Phase 2. Analyzing stakeholders’ needs.* One session was held in order to identify children’s needs and feelings when they are sick or in some type of pain. The objective of this activity was to get the children to empathize with the needs that they themselves or other children may have in case of hospitalization, as well as to identify how the children would respond to and solve the identified needs. To work on these objectives, children were asked to draw a sick child and to explain the drawing. Prior to the drawing activity, a group dynamics was conducted in which children were asked about their own or others’ experiences in hospitals. A discussion with the whole group was held in order to have the children empathize with hospitalized children according their own experiences of being ill.
- *Phase 3. Defining what the robot has to do.* Two sessions were planned to reflect on what kind of robot the children wanted to develop and why. The objective was to think about the main goal to be fulfilled by their social robot. The first activity consisted of thinking about objects that the pupils would bring with them to a hospital if they had to be hospitalized, including a description of the objects’ characteristics. This activity was carried out by means of a brainstorming and drawing session in groups of six pupils: each group had a paper mural and they were asked to draw whatever they wanted on it. The second activity consisted of a role-playing exercise where children were asked to play freely, accompanied by a series of objects, as if they were in a hospital.
- *Phase 4. Specifying the robot’s features.* Two sessions were held to design the robot’s form and function. The objective was to choose the robot’s pre-prototype’s appearance and functionalities. The activity, conducted over two days, began by organizing children in small groups of five (different groups from phase 3, which, this time, were organized by the teacher) that would make up a team for the design of the pre-prototypes. For these groups, different dynamics were organized in order to facilitate group debate and consensus in the drawing of their robots and the spelling out of the desired robot functionalities. The dynamics consisted of trying to find consensus on the decisions and helping the children to develop their ideas. No examples of existing robots were offered, nor of the possible functionalities already developed for social robots in children’s hospitals. The first activity finished with the drawing of their robots, and the second one with the specification, orally or in writing, of its features.
- *Phase 5. Developing prototypes.* During four sessions, each group built its social-robot prototype. The main goal of the first activity at this stage was to build a conceptual prototype in modelling clay (two sessions) on the basis of the drawings made in phase 4. In the second activity, the children built the same prototype with robotics construction blocks (two additional sessions) and chose one of the features defined in phase 4, the most relevant for them, to be programmed. For this, Lego Mindstorms EV3 construction blocks were used, along with the EV3 Programmer, a programming language that uses a building-block visual interface that allows children to stack together programming components, such as actions, events and operators. For the construction and programming of the block prototypes, each group received intensive help from a member of the research team, because, in some cases, it was still a difficult task for the children.
- *Phase 6. Validating the prototypes.* Two sessions were used to finish the process. The main objective was to simulate a validation process with their peers and the research group in order to systematize and reflect on the entire design process. In the first activity, groups exchanged prototypes and played with the construction-block prototypes designed by other groups. In the second activity, each group’s prototype was presented to the two coordinators of the research team (a social scientist and a roboticist).

## 3. Results

A thick description of the participatory process was produced to give significance to the children’s imaginaries of HRI generated during the three-month design process. Although the analyses of data were mostly qualitative, based on a field diary and video recordings, on some occasions numerical analysis of data was used, specifically to analyze the drawings made by the children after categorizing some of the elements that appeared in them.

Three particularities were identified in children’s imaginaries about interactions with a social robot for a children’s hospital. Two of them are connected with how children imagine interaction in a health context and with a social robot. The other one has to do with the particular context of the participatory process of design.

### 3.1. An Assemblage of Relations

Before deciding on what robot they wanted to build (phase 4), the children participated in two previous stages (phase 2 and phase 3) organized to place them in the context of the investigation. The aim of this previous work was to reflect collectively about the social significance of children’s healthcare environments, as much as about the situations and the needs of sick or hospitalized children, which make up the context that social robots for healthcare purposes are conceived for. Later on (phase 4), the form and functions of each robot would be decided upon on all these previous representations. The results that we present here as “*an assemblage of relations*” are the product of all of these three phases of the process, and their interpretation only gets its full sense from the accumulated experience. Thus, the results identified in phase 4 are inextricably linked to the process initiated in phase 2, and it is for this reason that they are presented in one single line of argument.

To work on the initial goal of identifying the needs of hospitalized or ill children (phase 2), the participant children were asked to make a drawing depicting “How I feel when I am sick”. After producing their drawings, the children explained to their groups who the people in the drawing were, what the setting was, etc.

The drawings were analyzed with respect to what they could show about the children’s representation of care when ill or hospitalized, and with respect to what tools or concepts were made apparent. For doing this, all the items that showed up in drawings were categorized (Table 2). A total of 29 drawings were categorized. The defining categories were:

- Emotion of the sick child: happiness, sadness, pain, lack of expression, etc. (one emotion or another was assigned depending on the child’s face).
- Presence of objects: furniture, medical equipment, toys, etc.
- Presence of other people (relatives, medical staff).
- Place where the child was (home, hospital, open space).

**Figure 1:**
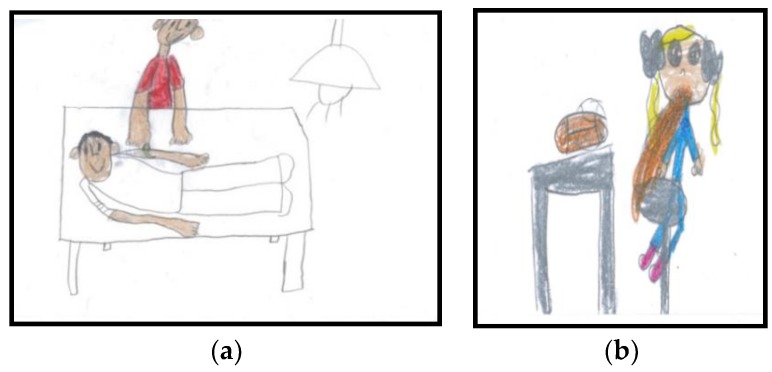
Drawings about “How I feel when I am sick” (phase 2): (**a**) Drawing in which the emotion of the sick child was categorized as happiness; (**b**) Drawing in which the emotion of the sick child was categorized as pain.

The analysis of the different elements in the drawings showed no relationship between the different emotions and the presence of things (TV sets, games, syrup, syringe, ball, flowers, plaster…) or the children’s locations (hospital, home, park). However, it is interesting that, in almost all the drawings expressing happiness, there were figures representing family members (72.7%) such as the mother, the father, and/or siblings (Figure 1a). Conversely, in almost all drawings that expressed sadness or pain, there was no other human figure (80%) (Figure 1b).

These results show how children represent themselves in a healthcare context and that these representations possess strong affective connotations. As the analysis of the drawings reveals, when children are sick or hospitalized, well-being (as shown in the pictures by the emotion of happiness in the child’s face) is associated with the presence of other people (i.e., relatives), and sadness is linked to the “non-presence” of other people.

Once the needs of ill and hospitalized children had been identified, the participants had to decide what kind of social robot they would build. This was done by defining what a social robot should do in a healthcare context (phase 3) and by specifying its appearance and functionalities (phase 4). To do so, the children, in groups of four to five students, were asked to develop a robot for a children’s hospital, decide its name and specify a set of characteristics for it, without any further indications. A total of six robots were conceived: Soft Bear, Stone Robot, Strange-Cat, Nolla Baby Tortoise, Noll Eagle and Robomama. These robots were associated with more than 50 different characteristics or features. In order to make sense of all these characteristics, they were classified into five broad categories (Table 3):

- Movement: walking, dancing, moving its ears, flying, swimming, turning somersaults…
- Care activities: taking care of you, kissing, hugging, feeding you, smelling like mum…
- Social abilities: playing instruments, hearing, thinking, laughing, crying, telling tales, telling jokes…
- Non-human stimulus-response: flashing when pressed or touched…
- Appearance elements: tail, soft, long hair, half boy-half girl, with wheels…

Although all the features that were identified mediate the relationship between humans and machines, it was considered that the most relevant characteristics for capturing the children’s imaginaries of HRI were those directly related to interaction, i.e., the ones categorized as: “Care activities”, “Social abilities” or “Non-human stimulus-response”. Almost all the interactive features imagined by the children made reference to human abilities which are meaningful in the assemblage of social interactions with other people, particularly with family members and medical personnel. The participant children had been able to define interactions with a robot in the framework of this social entanglement. They imagined their well-being when sick or hospitalized as an assemblage of caring relationships, as not being alone in a hospital (as was observed during phase 2). When they had been asked to imagine a robot for healthcare purposes, they had put it in this setting, and they had imagined relational properties as human-like properties. They had anthromorphized everything related to the robot: its setting, its appearance, its features and the interactions with it.

### 3.2. Bidirectional Care

**Figure 2:**
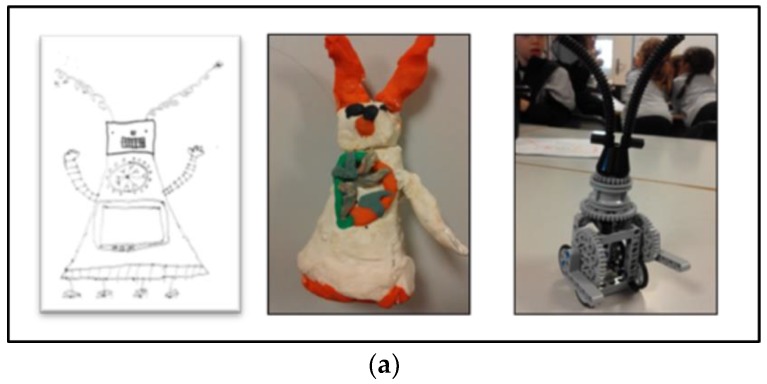

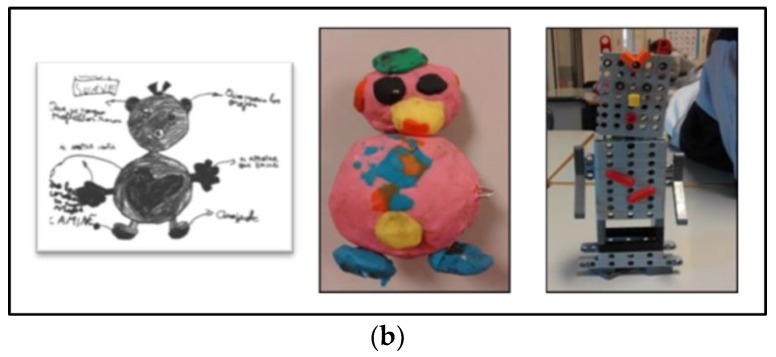
Examples of children’s prototypes developed from phase 4 to phase 5: (**a**) Stone Robot; (**b**) Soft Bear.

Once the needs of ill and hospitalized children had been identified, the participants in the design process had to decide what social robot they would build. This was done by defining what the robot had to do (phase 3) and specifying its appearance and functionalities (phase 4). More than obtaining information from the final social robots’ prototypes built by children (Figure 2), which were very basic, we focused on the building process.

We gathered our most relevant information while the children were playing with the prototypes as well as with their choice of features to be programmed into the robotics’ block prototype (phase 5). During the workshops devoted to prototyping with modelling clay and construction blocks (phase 5), children played with their robots plus some complementary elements conceived to take care of their social-care robot (companion pets, feeding utensils or costume accessories). These elements were built spontaneously on the children’s own initiative, and followed dynamics which had not been included in the guided participatory process for building robot prototypes. Below, we include some fragments of the children’s field diary explaining two episodes in which they added complementary elements in order to take care of their robots:

Episode “A pet for the robot”

Two children in the group are busy making the wings of the bird with modeling paste; they want the wings to be extended as if the bird is flying. They are trying to make them separately but, when they are hooked to the body, the wings break. Paul (fictional name) is not working on the wings or participating in the discussion. He is highly concentrated on a small piece of white modeling paste (like the eagle). He seems to be making a tiny eagle, modeling tiny wings coming out of both sides of its body. He uses two balls to make very small gray eyes, like eagle eyes. After a while, with downcast eyes, he says to one of the members of the research team that he needs orange modeling paste, because he needs it to finish the beak of the eagle and he needs just a small little bit. After a while, he shows the same researcher a little flat eagle (a two-dimensional eagle). Instead of a beak, it has two small orange balls in the tail and two more ones on the tip of each wing. Paul explains that it is the eagle’s pet for her to play with and keep her company—a small iguana.

Episode “Feeding the Stone Robot”

Of all the groups, only two do not design an anthropocentric non-mechanical figure. One of these groups created the Stone Robot, a robot with a type of geometry that is exactly the stereotype of an anthropocentric mechanical robot. The group is made up of four boys and one girl. They already finished their robot during the first session assigned to building the construction-block prototype. During the second session, the four boys and the girl walked through the classroom observing the work of other groups and playing with construction blocks. This is also what they did the following session, which was devoted to testing the prototypes. At the end of these two free sessions, they had put together some gadgets for their robot: a water bottle made with a large piece of construction block, a string found on the floor in class, and a pair of cupcakes made with two round pieces, one on top of the other, for when Stone Robot goes hiking.

In total, the children, grouped in subgroups of 4–5 students, designed six social-robot prototypes. For five of these prototypes, complementary elements for looking after the social robots were found (Table 4).

Predictably, since the children had been told to design a social robot for hospitalized children, all of them developed robots whose main feature consisted of an activity directly associated with care: with emotional care, such as kissing or singing a lullaby; or with entertainment, so that children would not be bored—in their own words, activities such as “walking or telling jokes”. When choisen features were impossible to programme, similar ones were programmed. For example, in the case of *Soft Bear*, instead of “smelling like a mother”, what was programmed was that, when you touched its hand, the previously recorded voice of the mother was heard. Only one mother’s voice was recorded; however, the children’s design included the feature that each robot would be personalized with the smell of the mother of the specific child it had to take care of.

As in a role-play activity, these elements reveal how children imagine and represent the significance of their caring relationships with the robot and the robot itself. Children designed the robots that had to take care of them as objects with some caring functionalities, such as kissing, singing a lullaby, shining for fun, etc. But they also designed things to take care of their robots, to feed them, to dress them and to keep them company, and they related to these robots as subjects in need of care. In their process of designing a social robot in case they were sick or hospitalized, children imagine their caring interactions in a bidirectional way: the robot takes care of them while they take care of the robot.

### 3.3. One Robot or Several Robots?

A total of six sessions were conducted with each class-group working on their prototypes: four prototyping sessions (two sessions for building a conceptual prototype with modelling clay and two sessions for building a functional one with robotics construction blocks) (phase 5), plus two additional sessions for testing them (phase 6). During all six sessions, the continuous construction/deconstruction process of prototyping was a constant mode of interaction with the robot. The process of construction/deconstruction was interpreted in the light of two different but complementary dynamics. One dynamic was associated with the development of the participatory process embedded in the daily dynamics of the school, and the other one was more connected with the children’s relation to the materiality of robots.

As it would be expected from any research or participatory process, the particular context and the conditions in which this one took place affected the final findings. Moreover, such findings took on a special meaning in that particular context. Obviously, since the participatory process was conducted at a school, its particular institution’s rules, norms and organization affected the processes. The most prominent effect of this influence was observed in connection with the process of care and maintenance of the prototypes. From one session to the other, the prototypes were kept on trays. Two trays were used for the modelling clay prototypes, and two more trays were later added to keep the robotics construction-block prototypes. During all six sessions, the course of the events was as follows. In the morning, a group of children would start an activity with their teachers and members of the project team and would give shape to a robot (first with modelling clay and then with robotics construction blocks). After the 50-min session, each group would leave their robot (more or less finished) on a tray that the teachers gave to them, together with the other robots built by the other groups. The trays, in the eyes of the children, would disappear from the classroom to be taken who knows where. The following week, on the morning of the same day of the week as the week before, the teachers of robotics and the members of the project team would come in again with the trays and the prototypes. Also, in the sessions for prototyping with construction blocks, the earlier modelling clay robots were always taken to the classroom (along with the construction-block ones) for the children to use them as references for their designed robots.

Intuitively, it could seem that the sessions would take up at the same point they had left off the week before. However, there is a relevant fact: when the trays left the classroom and disappeared from the eyes of children, they were kept in the school’s robotics room, a room with not much space and lots of things. In this room, the trays were stacked one on top of the other together with the trays of the other groups. The consequence was that, session after session, the modelling clay prototypes were progressively chopped, dismantled and dirtied. With the robotics construction-block trays, what happened was that, apart from the prototypes also being broken, the room where they were kept was the robotics room where older children went for their lessons and, when they needed some construction piece or programming bricks, they just took them from the trays. Thus, every week, on the day on which the participative process took place, the children had to devote some time to repair their prototypes as much as possible. Sometimes it was possible, but sometimes it was not, because the modelling clay was too dirty, or because a piece was missing and could not be replaced, or the brick had a different programming. Sometimes children were annoyed, or very annoyed; sometimes, they thought of an alternative; and still sometimes, the damages went unnoticed.

Apart from having to devote some of the initial time of each session to prototype-maintenance tasks, the children also related to their designed robots as if these were part of a ongoing open-ended process. Besides the planned activities that were proposed to them in each workshop, when children had completed their tasks with the prototype before the end of the session, when they became bored, or when, for whatever reason, they were not involved in the group task taking place at that moment, they continued making/re-making/un-making their prototypes. One fragment from the field diary describes this making/re-making/un-making process of prototyping:

Episode “Building and rebuilding the Strange-Cat”

On the first day devoted to the building of the block prototypes, the group that was designing the Strange-Cat finished building it. It was a group of five children (boys) who were highly motivated by the idea of designing a robot. On the second day, due to a relational problem among them, they were a bit upset at the start of the session. Informally, they had organized themselves into two subgroups of two and three children that worked separately. When they were given back their robot prototype made with construction blocks, the subgroup with three children demolished it and rebuilt it again with the same blocks, adding a longer piece as the robot’s body. The other subgroup of two made another block prototype of the Strange-Cat with the same form and a similar appearance, but with different colors and two horns more than the original design.

The participatory process and the imaginaries of HRI which were mobilized in the making of the social robots were inextricably embedded in the daily dynamics of the school where the project was carried out. In that context, storage limitations and the (scarce) attention of the research team to maintaining and caring for the children’s creations configured a setting in which the interactions with the robots were imagined as multiple relations with diverse robots. However, the very possibility of imagining a diversity of robots out of their single one was interpreted as a finding in itself, one that shows that the attachment of the children to the robots’ materiality during the participative process was a precarious one. The children imagined diverse potential interactions with their robots, which resulted in their making more than one actual robot.

## 4. Discussion

The results of the participatory experience were interpreted in light of some of the theses developed in the STS literature, particularly the ones that place care in the center of the debate about health technologies. The analysis of the imaginaries of HRI was very useful as a way of approaching ethical and social concerns when designing a social robot for a children’s hospital and improving the design process thanks to the involvement of the potential users from the earliest design stages, as demonstrated by previous research [47], because it ensures the inclusion of the users’ own views, experiences, and expertise.

As proposed in other similar studies about imaginaries of technologies [59], the analysis of imaginaries of HRI in healthcare explored children’s prospective representations concerning care and their relationship with social robots. This type of analysis gives us a better understanding of what good care means for the potential users of social robots in hospitals and allows for an interesting reflection on some assumptions made in previous child-robot interaction experiments [3,4,8,60,61]. In such scenarios, children are predominantly taken individually, and their interaction with previously designed robots is always conceived as isolated from adults.

According to the analysis we carried out of the children’s drawings, children define well-being in healthcare settings as associated with the possibility of relating to other people. Their representation of themselves alone while hospitalized or sick is a sad representation, while that of being surrounded by relatives, siblings or medical personnel is a happy one. Their prospective interactions with the robot are imagined in this context and are represented in a network of care relations. As with other care technologies, the inclusion of social robots in healthcare contexts takes place within an intricate mix of humans and things. As has been shown by assessments of the use of care technologies [62,63], artifacts or devices are embedded in a network of caring relationships among several actors. This is especially relevant when thinking about and designing the robot’s agency, and its ability to interact with children, to reduce their pain or anxiety or to make their hospitalization more pleasant. When introducing social robots in health contexts, we must be aware that children’s well-being is not only defined by the robot’s capacity to interact with them, but also by its capacity to interact with the child’s entire social system of care relationships and to be interacted by it.

This interpretation of the imaginaries of HRI has relevant implications for the conceptualization of social robots, as it opens up a dialogue with the results of child-robot interaction studies in hospital environments conducted with child-robot interaction experiments. In these experiments, a common concern is the potential problems connected with the robot’s behavior, for instance, if it stops moving or runs out of battery power. To solve these, sometimes an adult is involved in helping the child understand the situation and prevent a feeling of uncertainty. Nevertheless, this person is instructed to avoid interacting with the child or the robot during the experiment and to pretend not to be following the interaction. Thus, the presence of an adult is identified as a risk or a possible bias in the results, since it interferes with the “spontaneous” responses of children who, as opposed to adults, show greater trust in the robot itself [64]. Actually, it is considered that the greater number of times the child refers to the adult in the room for clarification, the less engagement with the robot [61]. However, based on the imaginaries identified in our experience, taking the presence of adults as a bias is quite odd, given the way children conceive of their possible experience in hospitals. The “spontaneous” representation of well-being of young children involves the company of adults, and their presence certainly mediates children’s relationships with other people or artifacts. In this sense, our findings only broaden the challenge proposed by Belpaeme et al. [4]. Against the classical perspective in HRI that understands social cognition as residing inside the agent, and based on studies conducted in hospital settings, some scholars [4] propose understanding social cognition as a continuous and self-correcting interaction between two agents. However, for interaction in a healthcare context to be meaningfully social for children, the design and the building of social robots has to integrate the presence of significant adults in the forming of interactions, which implies conceptualizing social cognition as an agency distributed among the network of actors present in healthcare environments.

In addition, when children imagine specific HRI in a healthcare setting, they attribute typical social behaviors to the robot. As we have previously shown, young children attribute the human qualities of cognition, affection or schemata to robots [4,60,65,66]. This can be interpreted in line with what is stated by Suchman [9] from an STS approach, namely, that social robots can be understood both as objects and subjects: as artifacts with their own functionalities (objects) and as representations of our own humanity (subjects). When, during the participatory process, children imagine and design additional elements for taking care of their robots (the robot as a subject), which have in turn been conceived for the purpose of taking care of them (the robot as an object), they imagine the HRI as a bidirectional relationship of care. When the aim of designing social robots for healthcare purposes is to foster social interaction between the robot and the child in order to improve well-being in hospital settings, it is important that the procedure for introducing the robot takes into account the anthropomorphizing tendency of children [61]. In this sense, integrating the possibility of bidirectional care in the creation of a social robot is understood as a way of introducing in the robot the child’s comprehension of their own caring relationships, and this can be especially useful, for example, in order to integrate the autonomy problems of robots in HRI in a positive way.

Finally, the dynamics observed during the process of building modelling clay and construction-block robots, a dynamics of constantly making/re-making/un-making the robot, calls into question the notion of HRI as a relation between one human and one robot. If, with the imaginary of an assemblage of relations, the notion of “the human” gets problematized, with the emergence of a network, the imaginary of several robots problematizes “the robot”. As stated by Corsín Jiménez [67], one of the main characteristics of prototypes is their capacity to call upon themselves, to re-functionalize their own purpose, producing new formats and devices for collective thinking in an ongoing open-ended process. This feature of prototypes was vividly identified during the process of robot design by children. Although prototypes are typically subject to processes of constant change, the storage limitations of the school only facilitated such change. The whole dynamics of putting away and maintaining the prototypes made of modelling clay and robotics construction blocks is closely connected with caring for the things which were being produced during the experience. Bringing such care issues to the fore is a way of acknowledging the overlooked dimensions of caring in material ordering [54,68]. The particular conditions for taking care of the robots during the participatory process account for the very emergence of multiple robots. As was observed throughout the design stages, both potentially and actually, the children’s imaginary of the interaction is not with one robot, but with several robots. Or, more accurately, it is the imaginary of several robots in the making.

This observed relationship could be used in designing the appearance of social robots in order to address what some researchers have identified as a loss of child engagement in long-term interactions [69]. This interpretation is in line with those that propose enhancing long-term social child-robot interactions with adaptive robots which are able to switch between multiple activities [3]. This would prevent children’s disengagement with robots due to, among other things, their high expectations regarding the robot’s behavior [4,61]. In the case of long hospitalizations or treatments, and in light of the children’s imaginaries of HRI while prototyping, besides developing the possibility of multiple activities [3], it is also important to explore the possibility of achieving engagement with one robot that could mobilize the emergence of diverse robots by integrating, for example, some kind of functional flexibility or transformative appearance.

## 5. Conclusions

The aim of this paper was to propose a new resource to reflect on the social and ethical issues related to social robots for children’s healthcare environments: the analysis of the imaginaries regarding HRI. Thick description of a participatory process conducted with six-year-old children made it possible to carry out the analysis of children’s imaginaries that emerge while they are designing a social robot, particularly those imaginaries of HRI linked to children’s meaning of care in healthcare environments. Drawing upon perspectives that advocate for a reciprocal and dynamic relationship between technologies and society from the initial stages of design, our study of HRI was based on prospective imaginaries.

We argue that the study of imaginaries is a valuable tool in order to approach HRI with children. Our analysis reinforced previous findings in this research field, such as the tendency of children to anthropomorphize robots or the need to introduce new perspectives in the conceptualization of social cognition, conceptualizations that understand cognition as a product of interactions. Furthermore, children-robot interactions while making/imagining a social robot constitute an open field full of possibilities that gives us an opportunity to gain insight into children’s values, needs and practices. Children’s conceptualization of HRI as a network of interactions with the presence of other significant persons, their bidirectional care relationships with robots, and their engagement with the machine through multiple potential robots are the major findings of this study. These representations about how care, or “good” care, is represented by children when they imagine themselves interacting with a social robot in healthcare environments could only be identified thanks to the observation of the robot-making process from its very inception.

## Figures and Tables

**Table 1:** Phases of the participatory process for the design a social robot with children.

Phases in the Design of A Social Robot	Objectives to be Reached with Children	Activities to Perform in the School
1. Organizing an interdisciplinary team	To share a workflow with engineers and social science researchers	What is an engineer? What does he/she do? (all group) 1 session
2. Analyzing stakeholders’ needs	To empathize with sick children’s needs	How do I feel when I’m sick? (individual) 1 session
3. Defining what we want the robot to do	To choose what robot we want to develop	What thing would I bring with me to a hospital? (small groups) Role-playing of a hospital (small groups) 2 sessions
4. Specifying the features (functional and of design)	To choose the appearance and functionality of the robot	Defining the robot’s appearance and its features (small groups) 2 sessions
5. Developing prototypes (conceptual and functional)	To build prototypes	Building prototypes with modelling paste (small groups) Building prototypes with robotics construction blocks (small groups) 4 sessions
6. Validating the prototype (fatigue tests/robustness and users)	To test prototypes	Playing with prototypes (small groups) Presenting prototypes (small groups) 2 sessions

Source: Authors’ own elaboration.

**Table 2:** Analysis of the drawings “How I feel when I am sick”.

Emotion on the Child’s Face	Total	Presence of People (*n*, %)	No Presence of People (*n*, %)
Happiness	11	8 (72.7)	3 (27.3)
Sadness & pain	10	2 (20.0)	8 (80.0)
Surprise	1	1 (100.0)	0 (0.0)
Lack of expression	7	2 (28.6)	5 (71.4)
Total	29	13 (44.9)	16 (55.2)

Source: Authors’ own elaboration.

**Table 3:** Analysis of the imagined social robots’ features.

Robots’ Features	Total (*n*, %)
Movement	16 (29.0)
Care activities	11 (20.0)
Social abilities	20 (36.4)
Non-human stimulus-response	2 (3.6)
Appearance elements	6 (10.9
Total	55 (100)

Source: Authors’ own elaboration.

**Table 4:** Robot’s caring characteristics as objects and subjects.

Social-Care Robot Prototype	Robot as Subject: *Complementary Care Element*	Robot as Object: *Choice Feature for Programming*
*Soft Bear*	Tie, flowers, buttons	Smelling like a mother
*Stone Robot*	Cupcakes, bottle of water	Singing a lullaby
*Strange-Cat*	Pizza	Walking
*Nolla Baby Tortoise*	-	Hiding and taking off head
*Noll Eagle*	Small pet	Telling jokes
*Robomama*	Tie, bag	Kissing

Source: Authors’ own elaboration.
